# TRIAGE Toolkit: Streamlined Discovery of Regulatory Genes and Elements

**DOI:** 10.1002/cpz1.70413

**Published:** 2026-07-01

**Authors:** Qiongyi Zhao, Sophie Shen, Yuliangzi Sun, Enakshi Sinniah, Mikael Boden, Nathan J. Palpant, Woo Jun Shim

**Affiliations:** ^1^ Institute for Molecular Bioscience The University of Queensland Brisbane Australia; ^2^ School of Chemistry and Molecular Biosciences The University of Queensland Brisbane Australia

**Keywords:** cell identity, regulatory elements, regulatory gene prioritization, TRIAGE toolkit

## Abstract

Efficient discovery of regulatory genes and elements is essential for understanding cell identity, differentiation, and disease mechanisms. The TRIAGE methods are a set of well‐established computational approaches that identify context‐specific regulatory genes and prioritize regulatory elements across the genome. Previous publications have described the development of these algorithms, their benchmarking, and biological applications. Here, we provide step‐by‐step protocols for applying the TRIAGE methods to identify regulatory drivers from diverse input types, including gene expression matrices, gene lists, and genomic loci. It covers analyses of both bulk and single‐cell RNA‐seq datasets and enables genome‐wide interrogation of regulatory elements at single‐base resolution. The analysis is efficient, typically requiring <30 min of computation time on a personal computer. In addition to the step‐by‐step description of the TRIAGE analysis workflow, we provide the TRIAGE toolkit, available as both an R package and a Python implementation, to support flexible and scalable regulatory analysis across platforms. © 2026 The Author(s). *Current Protocols* published by Wiley Periodicals LLC.

**Basic Protocol 1**: Prioritization of regulatory genes from bulk RNA‐seq data

**Basic Protocol 2**: Identification of cell populations and regulatory genes in single‐cell RNA‐seq data

**Basic Protocol 3**: Prioritization of regulatory long noncoding RNAs

**Basic Protocol 4**: Prioritization of functional genetic variants from eQTL data

**Alternate Protocol**: Python‐based implementation of the TRIAGE workflow for regulatory gene and element prioritization

**Support Protocol**: Preparing a normalized expression matrix from bulk RNA‐seq count data

## INTRODUCTION

Regulatory genes and elements play critical roles in shaping cellular identity and function during development and disease. However, standard analysis workflows, such as differential expression analysis of RNA‐seq data, have inherent limitations in uncovering the underlying regulatory mechanisms that drive these processes. Differential expression analysis prioritizes highly expressed or variable genes, which may include functionally passive downstream responders, while overlooking lower‐expressed but biologically crucial regulators, such as transcription factors (TFs) or non‐coding RNAs. While considerable progress has been made in regulatory gene analysis with tools, such as GENIE3 (Huynh‐Thu et al., 2010), GRNBoost2 (Moerman et al., 2019), Lisa (Qin et al., 2020), SCENIC (Aibar et al., 2017), and SCENIC+ (Bravo Gonzalez‐Blas et al., 2023), these methods primarily focus on TFs and their downstream targets. As a result, there remains an unmet need for tools that can systematically identify a broader spectrum of regulatory components, extending beyond TFs to include other regulatory elements involved in cellular regulation and decision‐making.

To address this analytical gap, we previously developed TRIAGE (Transcriptional Regulatory Inference Analysis of Gene Expression) (Shim et al., 2020). The method leverages consortium‐scale datasets of broad H3K27me3 domains across diverse cell types. Building on an observation that broad H3K27me3 domains preferentially deposit on regulatory loci (Carelli et al., 2017; Rehimi et al., 2016), TRIAGE quantifies epigenetic repressive tendency of genes. This repressive tendency score (RTS) serves as an effective proxy for the regulatory potential of genetic elements. The RTS is then incorporated into four discrete functional modules of TRIAGE: TRIAGEgene, TRIAGEcluster, TRIAGEparser, and TRIAGEccs. TRIAGEgene applies the RTS to calculate weighted gene expression, thereby prioritizing genes with high regulatory potential for a given cellular context of interest, while effectively filtering out highly expressed but non‐regulatory genes, such as structural or housekeeping genes (Shim et al., 2020). This module yields gene ranks by predicted regulatory importance as an output. TRIAGEcluster refines single cell clustering, improving the identification of cellular diversity in single‐cell RNA sequencing (scRNA‐seq) datasets (Sun et al., 2023). On the other hand, TRIAGEparser clusters genes into functionally similar groups by parsing gene‐gene relationships based on shared H3K27me3 patterns (Sun et al., 2023). These three modules are integrated into our TRIAGE R package to provide a user‐friendly platform for incorporating regulatory inference into standard gene expression analysis pipelines (Zhao et al., 2025).

Recent developments have further broadened the scope of TRIAGE to support genome‐wide analysis at single‐base resolution. This expansion has led to development of a new module TRIAGEccs (Sinniah et al., 2025), a tool capable of prioritizing regulatory elements, including novel genes and other genomic loci, thereby allowing unbiased discovery of regulatory loci across the genome. Unlike earlier methods that focus predominantly on TFs, the TRIAGE workflow identifies a broader spectrum of regulatory components, including non‐coding RNAs, signaling molecules, RNA‐binding proteins, and other regulatory elements. The TRIAGE toolkit is highly adaptable and integrates seamlessly with standard data processing pipelines, supporting applications from bulk and single‐cell RNA‐seq to the interrogation of noncoding regions and genetic variants.

**Figure 1:**
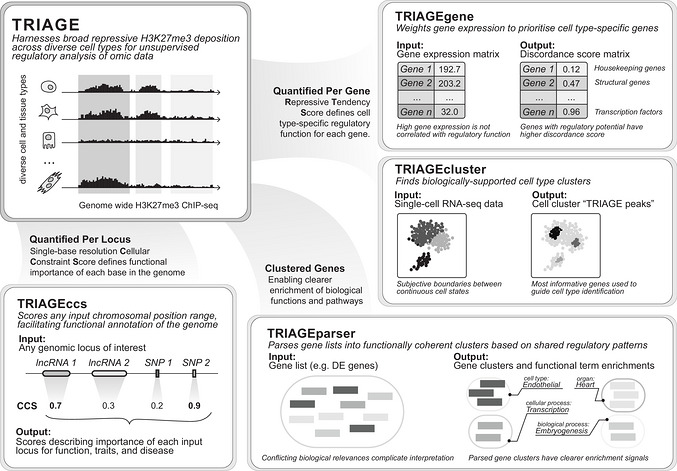
Overview of the TRIAGE toolkit. The TRIAGE toolkit enables unsupervised regulatory analysis of multi‐omics data by leveraging consortium‐scale H3K27me3 profiles across diverse cell types. It comprises four core modules: TRIAGEgene, TRIAGEcluster, TRIAGEparser, and TRIAGEccs, each supporting distinct data types and resolutions. TRIAGEgene accepts a normalized gene expression matrix and computes TRIAGE‐weighted discordance scores to prioritize genes with regulatory potential. TRIAGEcluster processes scRNA‐seq data to identify distinct cell type clusters, referred to as TRIAGE peaks. TRIAGEparser parses gene lists (e.g., differentially expressed genes) by grouping them into functionally coherent clusters based on shared regulatory patterns, enabling downstream functional enrichment analyses. TRIAGEccs performs single‐base resolution scoring of genomic loci from any input, e.g., genes, regulatory elements, or genetic variants, assigning a cellular constraint score that reflects regulatory importance at each genomic locus. Together, these components constitute a flexible and scalable framework for regulatory element prioritization and functional interpretation across complex biological systems.

In this article, we provide a step‐by‐step guide to the TRIAGE analysis workflow (Fig. 1). The workflow is designed to be accessible for researchers with limited computational experience, making it a practical toolkit for routine regulatory gene and element analysis. Specifically, we describe how each of the TRIAGE functional modules can be employed for different data modalities and analysis purposes in separate protocols. Implementation of TRIAGE for prioritization of regulatory genes (Basic Protocol 1), long non‐coding RNAs (Basic Protocol 3) and genetic variants (Basic Protocol 4) are discussed with illustrative examples. We also demonstrate utility of TRIAGE to identify and characterize a regulatory subset of cell populations (Basic Protocol 2). While the TRIAGE package was initially developed using R, we provide a complementary guidance for Python users in an Alternate Protocol.

## Basic Protocol 1 PRIORITIZATION OF REGULATORY GENES FROM BULK RNA‐SEQ DATA

**Figure 2:**
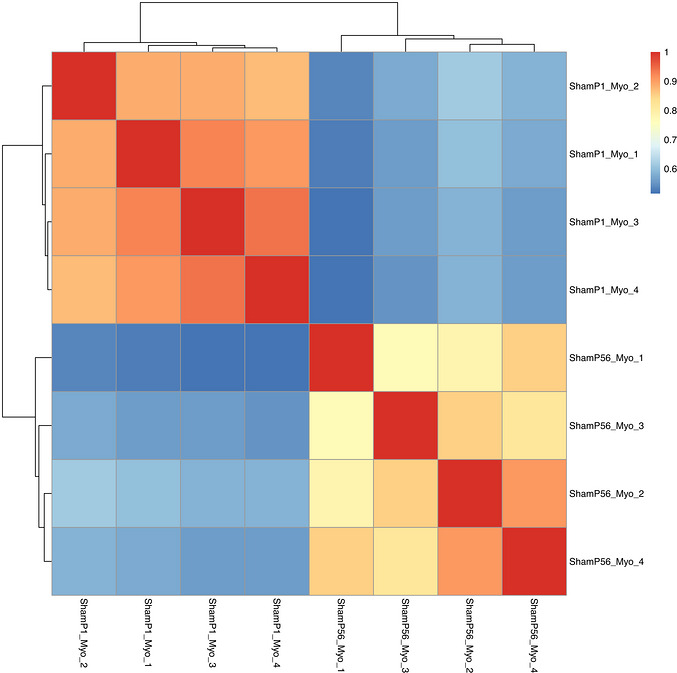
Jaccard similarity heatmap of Sham P1 and Sham P56 cardiomyocyte samples. Each condition includes 4 biological replicates. The heatmap was generated using the plotJaccard function from the TRIAGE R package.

In this protocol, we provide a detailed workflow for the prioritization of regulatory genes from bulk RNA‐seq data. A mouse bulk RNA‐seq dataset (Quaife‐Ryan et al., 2017) was used for demonstration (see Necessary Resources below). The analysis in R begins with loading the required libraries (step 1) and setting the working directory (step 2), followed by importing the input file as a normalized gene expression matrix or data frame (steps 3 to 6). TRIAGEgene is then applied to compute TRIAGE‐weighted expression or discordance scores (DS) for each gene (step 7). The results can be further analyzed by visualizing Jaccard similarity among biological replicates (Fig. 2; step 8), calculating mean DS per gene for each condition (step 9), and ranking genes by DS to assess regulatory potential (step 10).

### Necessary Resources

#### Hardware

Computer running Windows, Linux, or macOS

##### Software

R (https://cran.r‐project.org/)
  The code in this study was tested using R version 4.3.2; however, other versions are expected to be compatible.
RStudio Desktop (https://posit.co/downloads/)
  Although not required, this is recommended for an improved user interface and workflow.
TRIAGE toolkit (https://uniquest.store/product/triage2)

##### Files

Example input data
  In this protocol, we used a mouse bulk RNA‐seq dataset derived from purified heart cell populations of neonatal (P1) and adult (P56) mice. The dataset was obtained from the GEO database (Accession number GSE95755) and includes cardiomyocytes, fibroblasts, leukocytes, and endothelial cells isolated from infarcted and non‐infarcted hearts (Quaife‐Ryan et al., 2017). For demonstration, we focused on cardiomyocyte samples from Sham P1 and Sham P56 conditions.

1 Load required libraries.


R
# Load the TRIAGE R package
library(TRIAGE)
# Install the readxl package if not already installed, then load it.
# The readxl package is used to import Excel files.
if (!requireNamespace("readxl", quietly = TRUE)) {
install.packages("readxl")
}
library(readxl)

  The readxl R package is only required if Excel files need to be imported into R, as demonstrated in step 5.
2 Set working directory.


R
setwd("PATH/TO/YOUR/data")

3 Load the normalized gene expression data. Ensure that the input file is in the working directory specified in step 2.


R
file <‐ read.table("GSE95755_MultiCellularRNAseq_EdgeR_CPM.txt",
header=True, quote="", sep="\t")

4 Extract cardiomyocyte samples. In this example, Sham P1 and Sham P56 cardiomyocyte samples are compared; therefore, these samples are extracted from the input file, and the normalized gene expression data are stored in the variable “cpm”.


R
cpm <‐ file[,c("Symbol", "ShamP1_Myo_1", "ShamP1_Myo_2", "ShamP1_Myo_3",
"ShamP1_Myo_4", "ShamP56_Myo_1", "ShamP56_Myo_2",
"ShamP56_Myo_3", "ShamP56_Myo_4")]
rownames(cpm) <‐ file$Symbol
cpm <‐ cpm[,‐1]

5 Load differentially expressed genes (DEGs) from the input Excel file.


R
deg <‐ read_excel("GSE95755_MultiCellularRNAseq_AllSignificantRegulatedGenes.xlsx",
sheet = "ShP1vsShP56.Myo")

6 Extract normalized gene expression data for DEGs.


R
cpm_deg <‐ cpm[deg$Symbol, ]
# Save the file as a comma‐separated values (CSV) file
write.csv(cpm_deg, "ShP1vsShP56_DEG_CPM.csv", quote = FALSE, row.names = TRUE)

7 Run TRIAGEgene to generate TRIAGE‐weighted expression or DS values.


R
ds <‐ TRIAGEgene(cpm_deg, species = "Mouse")
# Save the TRIAGEgene result to a CSV file
write.csv(ds, "ShP1vsShP56_DS.csv", quote = FALSE, row.names = TRUE)

  Ensure that the input for “TRIAGEgene” is in the form of a data frame or matrix, and that the species is correctly specified (default: Human). It is recommended to provide normalized gene expression data that has been transformed using the natural logarithm. However, “TRIAGEgene” will automatically determine whether a log transformation is required based on the data characteristics, so users only need to ensure that the data is properly normalized. Alternatively, users can explicitly specify whether the input data has already been log‐transformed by setting the “log” parameter to “TRUE” or “FALSE” (e.g., “log = TRUE” or “log = FALSE”).
8 (Optional) Use the “plotJaccard” function to assess Jaccard similarity among biological replicates and generate a similarity heatmap (Fig. 2).


R
plotJaccard(ds, "Fig2_Jaccard_heatmap.pdf")

  Note that “ds” is the data frame generated in step 7, and “Fig2_Jaccard_heatmap.pdf” specifies the output file name.

9 Calculate the mean DS for P1 samples if key regulatory genes in P1 are of interest. The TRIAGE‐weighted DS can be used to rank genes according to their regulatory potential.


R
ds$ShamP1_Myo_DS <‐ rowMeans(ds[, c("ShamP1_Myo_1", "ShamP1_Myo_2",
"ShamP1_Myo_3", "ShamP1_Myo_4")])

  In this example, P1 samples have four biological replicates, named “ShamP1_Myo_1”, “ShamP1_Myo_2”, “ShamP1_Myo_3”, and “ShamP1_Myo_4”. The same approach can be applied to calculate the mean values across multiple replicates.
10 Parse the top ten TRIAGE‐ranked genes. In this example, all upregulated genes in Sham P1 were extracted first. Then, the top ten TRIAGE‐ranked genes were identified using the “topGenes” function (Table 1)


R
# Extract up‐regulated genes in Sham P1 samples
deg_upregulated <‐ deg[deg$logFC > 0, ]
ds_upregulated <‐ ds[deg_upregulated$Symbol, ]
# Identify the top ten genes with the highest DS values
top_genes <‐ topGenes(ds_upregulated, top_no = 10)
# Save the top ten genes to a file
write.table(top_genes[, "ShamP1_Myo_DS"],
"Table1_ShamP1_Myo_DS_top_genes.xls",
row.names = FALSE, col.names = FALSE, quote = FALSE)
# (Optional) Save all upregulated genes with their DS values to a tab‐delimited file
ds_table <‐ cbind(Gene_ID = rownames(ds_upregulated), ds_upregulated)
write.table(ds_table, "P1_upregulated_genes_DS.xls",
row.names = FALSE, quote = FALSE, sep = "\t")

  This will generate a ranked list of TRIAGE‐prioritized genes, including a table of the top ten genes (Table 1) and an optional complete list of upregulated genes with their DS values for downstream analysis. Higher DS values indicate greater regulatory potential as inferred by TRIAGE.

**Table 1:** Top Ten Genes Ranked by TRIAGE‐Weighted DS Values

Symbol	DS	Description
Sox17	0.972414	SRY‐box transcription factor 17
Wt1	0.814338	WT1 transcription factor
Hoxd8	0.777246	Homeobox D8 transcription factor
Ebf3	0.757584	EBF transcription factor 3
Igf2	0.739399	Insulin like growth factor 2
Sfrp1	0.677466	Secreted frizzled related protein 1
Foxc1	0.658488	Forkhead box C1 transcription factor
Ebf2	0.606114	EBF transcription factor 2
Rspo1	0.555276	R‐spondin 1
Nr2f2	0.490932	Nuclear receptor subfamily 2 group F member 2

## Basic Protocol 2 IDENTIFICATION OF CELL POPULATIONS AND REGULATORY GENES IN SINGLE‐CELL RNA‐SEQ DATA

**Figure 3:**
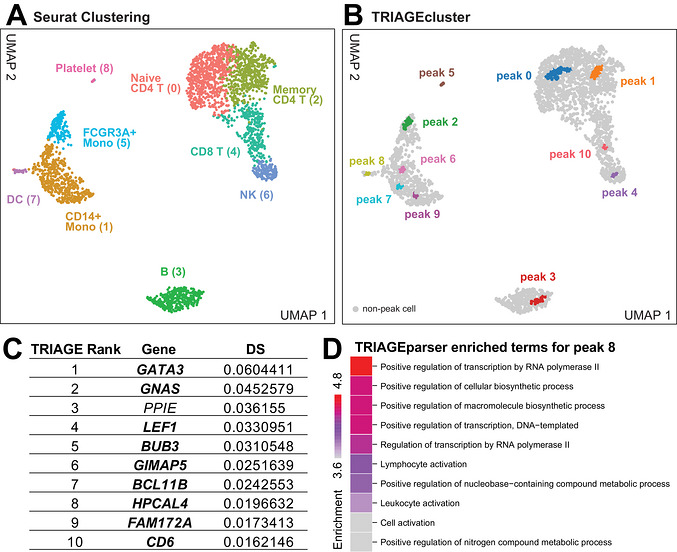
Application of TRIAGE to human scRNA‐seq data. (**A**) UMAP plot showing the PBMC dataset coloured by Seurat clustering and predefined cell type annotations. (**B**) UMAP plot showing TRIAGE peaks identified by TRIAGEcluster in the PBMC dataset. (**C**) Top 10 DS genes within TRIAGE peak 8, corresponding to dendritic cells (DC), identified using the topGenes function. Regulatory and/or signaling genes are indicated in bold italics. (**D**) Enriched STRING Gene Ontology terms identified by TRIAGEparser from the top 100 TRIAGE‐ranked genes within TRIAGE peak 8.

The TRIAGE toolkit can also be applied to single‐cell RNA sequencing (scRNA‐seq) datasets. Here, we use a publicly available human peripheral blood mononuclear cell (PBMC) dataset as an example to demonstrate the workflow. For scRNA‐seq analysis, a standard data processing workflow, such as Seurat (Hao et al., 2024) or Scanpy (Wolf et al., 2018) should first be performed; in this example, Seurat is used (steps 3 to 8). This workflow generates a UMAP for cell visualization (Fig. 3A) and produces a normalized gene expression matrix along with a metadata file. TRIAGEcluster is then used to identify TRIAGE peaks, representing distinct cell types or subtypes (Fig. 3B; step 9). Average expression within each peak‐defined cell population can be computed using the “byPeak” function, followed by application of TRIAGEgene to rank regulatory genes (Fig. 3C; steps 10 to 12). TRIAGEparser is subsequently used to parse gene groups within each cell group, perform STRING GO enrichment analysis, and visualize enriched terms in a heatmap (Fig. 3D; steps 13 to 14).

### Necessary Resources

#### Hardware

Computer running Windows, Linux, or macOS

##### Software

R (https://cran.r‐project.org/)
  The code in this study was tested using R version 4.3.2; however, other versions are expected to be compatible.
RStudio Desktop (https://posit.co/downloads/)
  Although not required, this is recommended for an improved user interface and workflow.
TRIAGE toolkit (https://uniquest.store/product/triage2)

##### Files

Example input data
  In this protocol, we used a publicly available PBMC scRNA‐seq dataset provided by 10X Genomics. This dataset, referred to as “pbmc3k”, contains 2700 single cells and is widely used for benchmarking single‐cell analysis pipelines. It can be downloaded directly from the 10X Genomics website, as described in step 2.

1 Load required libraries.


R
# Load the TRIAGE R package
library(TRIAGE)
# Install the required packages if they are not already installed, then load them
if (!requireNamespace("Seurat", quietly = TRUE)) install.packages("Seurat")
library(Seurat)
# Set seed for reproducibility
set.seed(88)

  The Seurat package is used for preprocessing, quality control, dimensionality reduction, clustering, and visualization of scRNA‐seq data.
2 Download and extract the publicly available PBMC 3k scRNA‐seq dataset from 10X Genomics.


R
url <‐ "https://cf.10xgenomics.com/samples/cell/pbmc3k/pbmc3k_filtered_gene_bc_matrices.tar.gz"
download.file(url, "pbmc3k_filtered_gene_bc_matrices.tar.gz")
untar("pbmc3k_filtered_gene_bc_matrices.tar.gz")

3 Load the PBMC dataset and create a Seurat object with basic filtering criteria to remove low‐quality cells and genes.


R
# Read 10X Genomics data
pbmc.data <‐ Read10X(data.dir = "filtered_gene_bc_matrices/hg19/")
# Create Seurat object with filtering parameters:
# Include features detected in at least 3 cells and cells where at least 200 features are detected
pbmc <‐ CreateSeuratObject(counts = pbmc.data, project = "pbmc3k", min.cells = 3, min.features = 200)

4 Standard Seurat analysis workflow.


R
# Quality control metrics
pbmc[["percent.mt"]] <‐ PercentageFeatureSet(pbmc, pattern = "^MT‐")
# Visualize QC metrics
VlnPlot(pbmc, features = c("nFeature_RNA", "nCount_RNA", "percent.mt"), ncol = 3)
# Filter cells based on QC metrics
pbmc <‐ subset(pbmc, subset = nFeature_RNA > 200 & nFeature_RNA < 2500 & percent.mt < 5)
# Normalize and scale data
pbmc <‐ NormalizeData(pbmc)
pbmc <‐ FindVariableFeatures(pbmc, selection.method = "vst", nfeatures = 2000)
all.genes <‐ rownames(pbmc)
pbmc <‐ ScaleData(pbmc, features = all.genes)
# Dimensionality reduction and clustering
pbmc <‐ RunPCA(pbmc, features = VariableFeatures(object = pbmc))
pbmc <‐ FindNeighbors(pbmc, dims = 1:10)
pbmc <‐ FindClusters(pbmc, resolution = 0.5)
pbmc <‐ RunUMAP(pbmc, dims = 1:10)

  Perform standard Seurat analysis workflow, including quality control, normalization, feature selection, scaling, dimensionality reduction, and clustering.
5 Data visualization using UMAP (Fig. 3A).


R
# Assigning cell type identity
new.cluster.ids <‐ c("Naive CD4 T (0)", "CD14+ Mono (1)", "Memory CD4 T (2)", "B (3)", "CD8 T (4)", "FCGR3A+ Mono (5)", "NK (6)", "DC (7)", "Platelet (8)")
names(new.cluster.ids) <‐ levels(pbmc)
pbmc <‐ RenameIdents(pbmc, new.cluster.ids)
# Generate UMAP plot (Fig. 3A)
pdf("Fig3a_PBMC_UMAP_cell_types.pdf", width = 6, height = 6)
p <‐ DimPlot(pbmc, reduction = "umap", label = FALSE, pt.size = 0.5) + NoLegend()
LabelClusters(p, id = "ident", repel = TRUE, box = TRUE, size = 2.5)
dev.off()

  Visualize cell populations using UMAP and annotate clusters based on known cell type markers.
6 Extract normalized expression data and save it to a CSV file.


R
all_genes <‐ as.data.frame(as.matrix(GetAssayData(pbmc, assay = "RNA", layer = "data")))
# Save the normalized expression data to a CSV file
write.csv(all_genes, "pbmc_allgenes.csv", quote = FALSE)

7 Extract metadata with UMAP coordinates and save it to a CSV file.


R
# Add UMAP coordinates to the metadata
pbmc_meta <‐ cbind(pbmc@meta.data, as.data.frame(Embeddings(pbmc[["umap"]])))
# Add cell barcode information as a new column
pbmc_meta$cell_name <‐ rownames(pbmc_meta)
# Save the metadata to a CSV file
write.csv(pbmc_meta, "pbmc_metadata.csv", quote = FALSE)

  Ensure that both the UMAP coordinates and cell barcode information are included in the metadata file, as these columns are required for running TRIAGEcluster.
8 (Optional) Save the Seurat object to an RDS file for later use.


R
saveRDS(pbmc, "pbmc_seurat_object.RDS")

  This step is optional. The saved Seurat object can be reused in future analyses by loading the RDS file using “readRDS()”.
9 Run TRIAGEcluster to identify TRIAGE peaks representing putative cell populations or subpopulations (Fig. 3B).


R
TRIAGEcluster(expr = "pbmc_allgenes.csv",
metadata = "pbmc_metadata.csv",
outdir = "TRIAGE_Cluster",
output_prefix = "TRIAGE_Cluster",
cell_column = "cell_name",
umap_column = "umap_",
seed = 88) # Optional: set seed for reproducibility

  Ensure that the correct cell identifier column (e.g., “cell_name” in this example) and UMAP coordinate prefix (e.g., “umap_”) are specified. By default, the parameters are set to cell_column = “Barcode” and umap_column = “UMAP_”. The first column of the expression data file (“pbmc_allgenes.csv”) must contain the same set of cell names as the “cell_name” column in the metadata file.
  The output from TRIAGEcluster comprises a series of UMAP plots generated at different bandwidth resolutions, along with corresponding metadata files for each bandwidth. Each TRIAGE peak on the UMAP plot represents a predicted cell population or subpopulation. Similar to clustering analysis using Seurat's “FindClusters” function, which requires the user to determine an appropriate resolution, the TRIAGE peaks produced by TRIAGEcluster should be examined to select the bandwidth resolution that best suits the analysis.
10 Calculate average expression for each TRIAGE peak with bandwidth 0.2 (bw = 0.2).


R
# Run the ‘byPeak’ function to calculate the average expression for each TRIAGE peak, defined by the ‘Peak’ column using the ‘peak_column’ parameter
peak_avg <‐ byPeak(expr = "pbmc_allgenes.csv",
peak = "TRIAGE_Cluster/TRIAGE_Cluster_bw0.20_metadata.csv",
peak_column = "Peak", cell_column = "cell_name")
# Save the peak‐level average expression data to a CSV file
write.csv(peak_avg, "Peak_AvgExp.csv", quote = FALSE)

  This step generates peak‐level average expression data stored in the “peak_avg” data frame and saved as a CSV file. These data can be used to characterize gene expression patterns within each TRIAGE peak and to facilitate downstream analysis of cell populations (e.g., cell types or cellular states).
11 (Optional) Calculate average expression for each Seurat cluster. The “byPeak” function can extract average expression for any group of cells from scRNA‐seq data and can therefore be applied to Seurat clusters or other groups of interest. Below, it is used to calculate average expression for each Seurat cluster.


R
# Run the ‘byPeak’ function to calculate average expression for each Seurat cluster
cluster_avg <‐ byPeak(expr = "pbmc_allgenes.csv",
peak = "pbmc_metadata.csv",
peak_column = "seurat_clusters",
cell_column = "cell_name")
# Save the cluster‐level average expression data to a CSV file
write.csv(cluster_avg, "Cluster_AvgExp.csv", quote = FALSE)

  This step is optional and demonstrates the flexibility of the “byPeak()” function. The function can be applied to any group of cells of interest by specifying the “peak_column” parameter, which enables calculation of average expression for user‐defined cell groupings.
12 Run TRIAGEgene on peak‐level average expression data (Fig. 3C).


R
# Run TRIAGEgene to calculate TRIAGE‐weighted DS values on peak‐level average expression data
ds <‐ TRIAGEgene(peak_avg)
# Save the TRIAGE‐weighted DS values to a CSV file
write.csv(ds, "Peak_DS.csv", quote = FALSE)
# Extract top 100 TRIAGE ranking genes for specific peak (e.g., Peak8)
sorted_indices <‐ order(ds$Peak8, decreasing = TRUE)
top_genes <‐ rownames(ds)[sorted_indices[1:100]]
# Save the top 100 genes in Peak8 to a tab‐delimited file
write.table(top_genes, file = "Peak8_top100genes.txt",
row.names = FALSE, col.names = FALSE, quote = FALSE, sep = "\t")
# Extract top 10 genes for all peaks
top_genes <‐ topGenes(ds, top_no = 10)
# Save results to a CSV file
write.csv(top_genes, "Peak_DS_top10.csv", quote = FALSE, row.names = FALSE)

  TRIAGEgene is applied to prioritize regulatory genes based on peak‐level average expression data. This step generates TRIAGE‐weighted discordance scores for each gene across TRIAGE‐defined cell populations. The results include ranked gene lists for individual peaks (e.g., Peak8) as well as the top‐ranked genes across all peaks. Figure 3C illustrates the top ten TRIAGE‐ranked genes in TRIAGE peak 8.
13 Run TRIAGEparser on top 100 genes from TRIAGE peak 8 and visualize the GO enrichment results using the “plotGO” function (Fig. 3D).


R
# Run TRIAGEparser for single peak analysis
TRIAGEparser("Peak8_top100genes.txt", outdir = "TRIAGE_Parser_Peak8")
# Run the plotGO function to visualize enriched STRING Gene Ontology terms in top 100 TRIAGE ranked genes in TRIAGE peak 8 (Fig. 3d)
plotGO(indir = "TRIAGE_Parser_Peak8", outdir = "TRIAGE_Parser_Peak8")

  TRIAGEparser is applied to identify enriched biological pathways for the top 100 TRIAGE‐ranked genes in TRIAGE peak 8. The results are visualized using the plotGO function, which displays enriched STRING Gene Ontology terms (Fig. 3D). Internet access is required for this step, as TRIAGEparser queries the STRING database for GO enrichment analysis.
14 Run TRIAGEparser on the top 100 genes for each TRIAGE peak.


R
# Run TRIAGEparser for all peaks
TRIAGEparser("Peak_DS.csv",
input_type = "table",
outdir = "TRIAGE_Parser",
number_of_gene = 100)
# Run plotGO to visualize enriched STRING Gene Ontology terms in gene clusters identified by TRIAGEparser
plotGO(indir = "TRIAGE_Parser", outdir = "TRIAGE_Parser")

  For TRIAGEparser, when the input type is set to “table”, the function iterates through each column of the input table (where each column, starting from column 2, represents a TRIAGE peak in this example). Genes are ranked based on TRIAGE‐weighted discordance scores, and the top genes (as specified by the “number_of_gene” parameter; here, top 100) are extracted for downstream pathway enrichment analysis.

This protocol demonstrates a workflow for processing scRNA‐seq data by integrating the TRIAGE toolkit with the Seurat analysis framework. TRIAGEcluster is used to refine cell population structure, followed by TRIAGEgene to prioritize regulatory genes and TRIAGEparser to identify enriched biological functions. The workflow generates TRIAGE‐based regulatory gene rankings for each identified cell population, along with gene sets enriched for GO terms (Fig. 3). The results include ranked gene lists for individual TRIAGE peaks or Seurat clusters, top candidate regulatory genes, and GO enrichment summaries, enabling interpretation of cell‐type‐specific regulatory programs and supporting downstream functional analysis.

## Basic Protocol 3 PRIORITIZATION OF REGULATORY LONG NONCODING RNAS

The TRIAGE can prioritize not only known genes from bulk and single‐cell RNA‐seq datasets, but also novel genes, long noncoding RNAs (lncRNAs), and other genomic loci of interest. In this protocol, we demonstrate the application of the TRIAGE toolkit to prioritize regulatory lncRNAs. LncRNA data are first obtained from the LNCipedia database (Volders et al., 2013). TRIAGEccs is then used to compute cellular constraint scores (CCS), which is an equivalent term for RTS used to study whole genome at a base‐pair resolution, for each lncRNA across the genome (step 3). Optionally, CCS can be computed for exon‐only regions by setting the “exons_only” parameter to “TRUE” (step 4). Candidate lncRNAs are ranked in a descending order of CCS values (step 5). Finally, inflection point analysis is performed using the inflection R package to define a threshold (step 6), and lncRNAs with CCS values above this threshold are considered high‐priority regulatory candidates.

### Necessary Resources

#### Hardware

Computer running Windows, Linux, or macOS

##### Software

R (https://cran.r‐project.org/)
  The code in this study was tested using R version 4.3.2; however, other versions are expected to be compatible.
RStudio Desktop (https://posit.co/downloads/)
  Although not required, this is recommended for an improved user interface and workflow.
TRIAGE toolkit (https://uniquest.store/product/triage2)

##### Files

Example input data
  lncRNA data were obtained from the LNCipedia database (Volders et al., 2013) (https://lncipedia.org/, version 5.2, hg38 build). For demonstration, we used all lncRNAs located on human chromosome 12. Details on data retrieval and extraction are provided in the protocol (step 2).

1 Load required libraries.


R
# Load the TRIAGE R package
library(TRIAGE)
# Install the inflection package if not already installed, then load it.
# The inflection package is used for inflection point analysis in Step 6.
if (!requireNamespace("inflection", quietly = TRUE)) {
install.packages("inflection")
}
library(inflection)

  Note that the inflection R package is only required in step 6 to identify the inflection point and is not necessary for routine use of the TRIAGE toolkit.
2 Download the LNCipedia lncRNA dataset and extract lncRNAs located on chromosome 12.


bash
# Download the lncRNA BED file from the LNCipedia database
wget ‐c https://lncipedia.org/downloads/lncipedia_5_2/full‐database/lncipedia_5_2_hg38.bed
# Extract a subset of lncRNAs located on chromosome 12 for demonstration
awk '$1=="chr12"' lncipedia_5_2_hc_hg38.bed > lncipedia_5_2_hc_hg38.chr12.bed

3 Run TRIAGEccs to calculate CCS values for all lncRNAs in chromosome 12.


R
ccs_whole <‐ TRIAGEccs("lncipedia_5_2_hc_hg38.chr12.bed",
rts_genome = "PATH/TO/TRIAGE_hg38.bedgraph",
output = "chr12_CCS_whole.bed")

  Since the LNCipedia dataset uses the hg38 genome build, the corresponding bedgraph file “TRIAGE_hg38.bedgraph” should be used. If the input BED file uses hg19 coordinates, the file should be replaced with “TRIAGE_hg19.bedgraph”. The corresponding RTS genome files can be downloaded from: https://uniquest.store/product/triage2.
4 (Optional) Calculate CCS for exon‐only regions by setting the “exons_only” parameter to TRUE.


R
ccs_exon <‐ TRIAGEccs("lncipedia_5_2_hc_hg38.chr12.bed",
rts_genome = "PATH/TO/TRIAGE_hg38.bedgraph",
output = "chr12_CCS_exon.bed",
exons_only = TRUE)

5 Rank lncRNAs by CCS values to prioritize those with higher regulatory potential.


R
# Sort lncRNAs in descending order of CCS
ccs_whole_sorted <‐ ccs_whole[order(ccs_whole$CCS, decreasing = TRUE), ]
# Save the ranked lncRNAs to a tab‐delimited file
write.table(ccs_whole_sorted, file = "LNCipedia_chr12_CCS.txt",
row.names = FALSE, col.names = TRUE, quote = FALSE, sep = "\t")
# Display the top 20 lncRNAs with highest CCS values
head(ccs_whole_sorted, 20)[,c(1:4,13)]

6 Identify statistically significant high‐CCS lncRNAs using inflection point analysis.


R
# Determine the inflection point in the ranked CCS list
inflection_whole <‐ uik(1:nrow(ccs_whole_sorted), ccs_whole_sorted$CCS)
# Display the identified inflection point
print(paste("Inflection point:", inflection_whole))
# Extract lncRNAs with CCS values above the inflection point
high_ccs_whole <‐ ccs_whole_sorted[1:inflection_whole, ]

  This protocol demonstrates a workflow for prioritizing regulatory long noncoding RNAs using the TRIAGE toolkit. TRIAGEccs is applied to compute cellular constraint scores (CCS) across genomic regions, enabling genome‐wide prioritization of lncRNAs based on their regulatory potential. The workflow generates ranked lncRNA lists, from which high‐priority regulatory candidates can be identified. These results facilitate the identification of functionally relevant lncRNAs and support downstream experimental validation.

## Basic Protocol 4 PRIORITIZATION OF FUNCTIONAL GENETIC VARIANTS FROM eQTL DATA

In this protocol, we highlight the applicability of TRIAGE toolkit at single‐base resolution. This protocol demonstrates the application of TRIAGE toolkit to prioritize functional genetic variants using human expression quantitative trait loci (eQTL) data from the GTEx project (Consortium, 2020). The GTEx eQTL data are obtained and imported into R (steps 30 to 31), then converted into BED format (step 32). The resulting BED file is analyzed using TRIAGEccs to compute CCS for each variant (step 33). To identify high‐priority regulatory variants, variants are ranked in a descending order of their CCS values (step 34). Inflection point analysis is used to define a threshold for high‐CCS variants (step 35), which are separated from low‐CCS variants for comparison (step 36). Finally, statistical comparisons between high‐ and low‐CCS variant groups are performed using Wilcoxon rank‐sum tests (steps 37 to 38).

### Necessary Resources

#### Hardware

Computer running Windows, Linux, or macOS

##### Software

R (https://cran.r‐project.org/)
  The code in this study was tested using R version 4.3.2; however, other versions are expected to be compatible.
RStudio Desktop (https://posit.co/downloads/)
  Although not required, this is recommended for an improved user interface and workflow.
TRIAGE toolkit (https://uniquest.store/product/triage2)

##### Files

Example input data
  In this protocol, we used eQTL data from the GTEx project, downloaded from the GTEx Portal (Consortium, 2020) (https://www.gtexportal.org/home/). Data from the left ventricle of the human heart were used for demonstration, and detailed instructions for data download and extraction are provided in step 2.

1 Load required libraries.


R
library(TRIAGE)
# Install the inflection package if not already installed, then load it.
# The inflection package is used for inflection point analysis in Step 6.
if (!requireNamespace("inflection", quietly = TRUE)) {
install.packages("inflection")
}
library(inflection)

2 Download GTEx eQTL data.


bash
# Download GTEx eQTL data (v8) from the GTEx Portal (https://www.gtexportal.org/home/)
wget ‐c https://storage.googleapis.com/adult‐gtex/bulk‐qtl/v8/single‐tissue‐cis‐qtl/GTEx_Analysis_v8_eQTL.tar
# Extract all files from the downloaded tar archive
tar xvf GTEx_Analysis_v8_eQTL.tar

3 Load the heart left ventricle data for demonstration.


R
# Use Heart_Left_Ventricle data for demonstration
input_file <‐ " GTEx_Analysis_v8_eQTL /Heart_Left_Ventricle.v8.signif_variant_gene_pairs.txt.gz"
# Load GTEx eQTL data
eqtl_data <‐ fread(input_file)

4 Convert GTEx variant to BED format with essential columns, including chromosome, start, end, and a unique ID. Additional annotations, such as nominal *p*‐value, effect size, and minor allele frequency (MAF), are not required for TRIAGE analysis but can be retained for downstream statistical comparisons.


R
# Parse variant coordinates from the 'variant_id' column and convert to BED format. An example of a variant ID is 'chr1_64764_C_T_b38', which includes the chromosome, position, reference allele, alternative allele, and genome build version.
eqtl_data[, c("chr", "pos", "ref", "alt", "build") := tstrsplit(variant_id, "_")]
# Construct BED coordinates and annotation columns, including chromosome, start position, end position, unique ID, nominal p‐value, effect size, minor allele frequency (MAF), and tissue type.
eqtl_data[, `:=`(
chr = chr,
start = as.numeric(pos) ‐ 1, # BED format is 0‐based
end = as.numeric(pos),
unique_id = paste0(variant_id, "‐", gene_id),
pval = pval_nominal,
effect_size = abs(slope),
maf = maf,
tissue = "Heart_Left_Ventricle")]
# Extract BED‐formatted data
bed_data <‐ eqtl_data[, .(chr, start, end, unique_id, pval, effect_size, maf)]
# Sort variants by chromosome and position
bed_data <‐ bed_data[order(chr, start)]
# Write to BED file
output_bed <‐ "Heart_Left_Ventricle_variants_v8_signif.bed"
fwrite(bed_data, output_bed, sep = "\t", col.names = FALSE)

  Note that only the first three columns (chromosome, start, end) are required for TRIAGEccs analysis. If the fourth column “unique_id” is not provided, TRIAGEccs will automatically generate a unique ID for each genomic locus. In this example, the required columns and a “unique_id” column are included. Additional columns (p‐value, effect size, and MAF) are optional, but p‐value and effect size can be used for downstream statistical comparisons of variant characteristics in steps 9 to 10.
5 Run TRIAGEccs to compute CCS values for each variant.


R
variants_ccs <‐ TRIAGEccs("Heart_Left_Ventricle_variants_v8_signif.bed",
rts_genome = "PATH/TO/TRIAGE_hg38.bedgraph",
output = "Heart_Left_Ventricle_variants_CCS.v8.all.bed")

6 Rank eQTL variants by CCS values.


R
# Rename unnamed columns to full descriptive names: nominal p‐value, effect size, and minor allele frequency
setnames(variants_ccs, old = c("V5", "V6", "V7"), new = c("pval", "effect_size", "maf"))
# Filter variants with CCS > 0 and sort in descending order
variants_ccs <‐ variants_ccs[CCS > 0]
ccs_sorted <‐ variants_ccs[order(‐CCS)]

7 Identify high‐CCS variants using inflection point analysis.


R
# Identify the inflection point
inflection_point <‐ uik(1:nrow(ccs_sorted), ccs_sorted$CCS)

8 Extract high‐ and low‐CCS variant sets for downstream comparison.


R
# Extract high‐CCS variants
high_ccs <‐ ccs_sorted[1:inflection_point, ]
# Extract low‐CCS variants
low_ccs <‐ ccs_sorted[(inflection_point + 1):nrow(ccs_sorted), ]

9 Compare nominal p‐values between high‐ and low‐CCS variants using the Wilcoxon rank‐sum test.


R
# Compare nominal p‐values between high‐ and low‐CCS variants
pval_test <‐ wilcox.test(high_ccs$pval, low_ccs$pval, alternative = "two.sided")
# Display results
cat("High CCS mean ‐log10(p‐value):", round(mean(‐log10(high_ccs$pval), na.rm = TRUE), 2), "\n")
# High CCS mean ‐log10(p‐value): 14.39
cat("Low CCS mean ‐log10(p‐value):", round(mean(‐log10(low_ccs$pval), na.rm = TRUE), 2), "\n")
# Low CCS mean ‐log10(p‐value): 10.45
cat("Wilcoxon test p‐value:", format.pval(pval_test$p.value, digits = 3), "\n")
# Wilcoxon test p‐value: <2e‐16

10 Compare effect sizes between high‐ and low‐CCS variants using the Wilcoxon rank‐sum test.


R
# Compare effect sizes between high‐ and low‐CCS variants
effect_test <‐ wilcox.test(high_ccs$effect_size, low_ccs$effect_size, alternative = "two.sided")
# Display results
cat("High CCS mean effect size:", round(mean(high_ccs$effect_size, na.rm = TRUE), 4), "\n")
# High CCS mean effect size: 0.4811
cat("Low CCS mean effect size:", round(mean(low_ccs$effect_size, na.rm = TRUE), 4), "\n")
# Low CCS mean effect size: 0.3776
cat("Wilcoxon test p‐value:", format.pval(effect_test$p.value, digits = 3), "\n")
# Wilcoxon test p‐value: <2e‐16

This protocol demonstrates a workflow for prioritizing functional genetic variants from eQTL data using the TRIAGE toolkit at single‐base resolution. The outputs include: (i) CCS‐annotated variant datasets, (ii) ranked lists of variants based on regulatory potential, and (iii) high‐ and low‐CCS variant groups defined by inflection point analysis. These results support the identification of putative functional regulatory variants for further biological interpretation and validation.

## PYTHON‐BASED IMPLEMENTATION OF THE TRIAGE WORKFLOW FOR REGULATORY GENE AND ELEMENT PRIORITIZATION

To enhance usability across programming environments, the TRIAGE toolkit provides both R and Python implementations. In addition to the R implementation, a Python‐based workflow is provided as an alternative for performing the TRIAGE analyses described in the four case studies above.

### Necessary Resources

#### Hardware

Computer running Windows, Linux, or macOS

##### Software

Python (https://www.python.org/)
  The code in this study was tested using Python 3.12.9; other Python versions ≥3.9 are expected to be compatible.
bedtools (https://bedtools.readthedocs.io/en/latest/content/installation.html)
  In this study, bedtools version 2.27.1 was used; other versions are expected to be compatible.
TRIAGE toolkit (https://uniquest.store/product/triage2)
  The downloaded TAR archive should be extracted, and the Python scripts can be found in the “TRIAGE/inst/python/” directory.
Python libraries
  The Python implementation of the TRIAGE toolkit requires the following packages: pandas, scipy, matplotlib, requests, scikit‐learn, and seaborn. These can be installed using conda (recommended):


bash
# To install all required Python libraries using conda:
$ conda install pandas scipy matplotlib requests scikit‐learn seaborn

##### Files

Example input data
  The input data used in this Alternate Protocol are the same as those described in Basic Protocols 1 to 4.

1 Run TRIAGEgene with the CSV input file from Basic Protocol 1, specifying the output file name and species. We recommend using the full path to TRIAGEgene.py.


bash
$ python PATH/TO/TRIAGEgene.py --input ShP1vsShP56_DEG_CPM.csv --output ShP1vsShP56_DS.csv --species Mouse

  For example, if “TRIAGEgene.py” is in “/home/tools/TRIAGE/inst/python/”, the command would be:


bash
$ python /home/tools/TRIAGE/inst/python/TRIAGEgene.py --input ShP1vsShP56_DEG_CPM.csv --output ShP1vsShP56_DS.csv --species Mouse

  This command generates an output file “ShP1vsShP56_DS.csv” in which gene expression values are transformed into TRIAGE‐weighted DS. These DS values can then be used to rank genes in descending order according to their regulatory potential.
2 Run TRIAGEcluster to generate TRIAGE peaks from scRNA‐seq data used in Basic Protocol 2 (Fig. 3B). For scRNA‐seq analysis, a standard preprocessing workflow should first be completed (see Basic Protocol 2, steps 3 to 8). After preprocessing, TRIAGEcluster can be executed using the following command:


bash
$ python PATH/TO/TRIAGEcluster.py --expr pbmc_allgenes.csv --metadata pbmc_metadata.csv --outdir TRIAGE_Cluster --cell_column cell_name --umap_column umap_ --seed 88

3 Run TRIAGEgene on peak‐level gene expression data.


bash
$ python PATH/TO/TRIAGEgene.py Peak_AvgExp.csv --output Peak_DS.csv

4 Run TRIAGEparser on top 100 genes from TRIAGE peak 8.


bash
$ python PATH/TO/TRIAGEparser.py --input Peak8_top100genes.txt --outdir TRIAGE_Parser_Peak8

5 Run TRIAGEccs to calculate CCS values for all lncRNAs in chromosome 12.


bash
$ python PATH/TO/TRIAGEccs.py ‐i lncipedia_5_2_hc_hg38.chr12.bed ‐o chr12_CCS_whole.python.bed ‐r PATH/TO/TRIAGE_hg38.bedgraph

  The input file “lncipedia_5_2_hc_hg38.chr12.bed” was generated in Basic Protocol 3.
6 Calculate CCS for exon regions by setting the “exons_only” parameter to TRUE.


bash
$ python PATH/TO/TRIAGEccs.py ‐i lncipedia_5_2_hc_hg38.chr12.bed ‐o chr12_CCS_whole.python.bed ‐r PATH/TO/TRIAGE_hg38.bedgraph --exons_only T

7 Run TRIAGEccs to compute CCS values for GTEx eQTL variants.


bash
$ python PATH/TO/TRIAGEccs.py ‐i Heart_Left_Ventricle_variants_v8_signif.bed ‐o Heart_Left_Ventricle_variants_CCS.v8.all.bed ‐r PATH/TO/TRIAGE_hg38.bedgraph

The input file “Heart_Left_Ventricle_variants_v8_signif.bed” was generated in Basic Protocol 4.

This Alternate Protocol provides a Python‐based implementation of the TRIAGE workflow, enabling regulatory gene and element prioritization across multiple data types. The workflow generates TRIAGE‐weighted expression or discordance scores for gene prioritization, identifies cell populations through TRIAGEcluster, and computes CCS for genome‐wide regulatory element analysis. The outputs include ranked gene lists, TRIAGE‐defined cell populations, and CCS‐annotated lncRNAs and genetic variants, which can be used for downstream functional interpretation, statistical analysis, and experimental validation.

## PREPARING A NORMALIZED EXPRESSION MATRIX FROM BULK RNA‐SEQ COUNT DATA

In Basic Protocol 1, normalized gene expression data were downloaded directly from GEO GSE95755. In some cases, however, users may only have access to gene‐level raw count data generated from bulk RNA‐seq experiments. This Support Protocol provides a practical workflow for preparing a normalized expression matrix suitable for TRIAGEgene analysis. The workflow starts from a raw count matrix, filters lowly expressed genes, performs library‐size normalization using edgeR (Chen et al., 2025), and generates a CPM‐normalized expression matrix that can be used as input for the TRIAGE toolkit.

### Necessary Resources

#### Hardware

Computer running Windows, Linux, or macOS

##### Software

R (https://cran.r‐project.org/)
  The code in this study was tested using R version 4.3.2; however, other versions are expected to be compatible.
RStudio Desktop (https://posit.co/downloads/)
  Although not required, this is recommended for an improved user interface and workflow.
TRIAGE toolkit (https://uniquest.store/product/triage2)
edgeR, available from Bioconductor
  This package is used for filtering lowly expressed genes and generating CPM‐normalized expression values.
limma (Ritchie et al., 2015), available from Bioconductor
  This is only required if batch correction is performed.

##### Files

Example input data
  This Support Protocol requires two input files: a gene‐level raw count matrix and a sample metadata file. The raw count matrix should contain genes as rows and samples as columns. Gene symbols should be provided in the first column. The metadata file should contain samples as rows and sample‐level information, such as condition and batch, as columns. The sample names in the first column of the metadata file must match the column names in the raw count matrix. Example input files are available on GitHub at: https://github.com/Qiongyi/TRIAGE_R_Package_v2/tree/main/protocol.

1 Load required packages.


R
# Install Bioconductor packages if required
# refer to https://bioconductor.org/packages/release/bioc/html/edgeR.html
if (!requireNamespace("BiocManager", quietly = TRUE)) {
install.packages("BiocManager")
}
# install edgeR if required
BiocManager::install("edgeR")
# Load edgeR
library(edgeR)

  The edgeR package is used to filter lowly expressed genes, calculate normalization factors, and generate CPM‐normalized expression values.
2 Load the raw count matrix and sample metadata.


R
# Load a gene‐level raw count matrix
# Rows should be genes and columns should be samples
counts <‐ read.table("raw_counts.txt", header = TRUE, sep = "\t", row.names = 1)
# load sample metadata
# Rows should be samples, and columns should contain sample‐level information
metadata <‐ read.table("metadata.txt", header = TRUE, sep = "\t", row.names = 1)

  The row names of metadata should match the column names of counts. If batch information is available, it should be included in the sample metadata file. See “metadata_with_batch.txt” for an example.
3 Check and match sample names between the count matrix and metadata.


R
# Reorder count matrix columns to match the sample order in metadata
counts <‐ counts[, rownames(metadata), drop = FALSE]

  This step ensures that the samples in the count matrix are in the same order as the rows in the metadata file. This is important because downstream functions use the condition and batch information in metadata.
4 Create an edgeR DGEList object.


R
# Create a DGEList object from the raw count matrix
dge <‐ DGEList(counts = counts)

5 Filter lowly expressed genes.


R
# Filter lowly expressed genes
keep <‐ filterByExpr(dge, group = metadata$condition)
# Retain expressed genes and reset library sizes
dge <‐ dge[keep, , keep.lib.sizes = FALSE]

  This step removes genes with little or no expression across the analyzed samples. This improves downstream analysis by reducing noise from uninformative genes.
  If condition information is not available, users can apply expression filtering without specifying a group:


R
keep <‐ filterByExpr(dge)
dge <‐ dge[keep, , keep.lib.sizes = FALSE]

6 Calculate normalization factors.


R
# Calculate normalization factors
dge <‐ calcNormFactors(dge)

  This step calculates sample‐specific normalization factors using the trimmed mean of M‐values (TMM) method implemented in edgeR. Normalization factors account for differences in library size and RNA composition between samples, which helps make expression values comparable across samples.
7 Generate CPM‐normalized expression values when batch correction is not required.


R
# Generate CPM‐normalized expression values
cpm_norm <‐ cpm(dge, log = FALSE)
# Add gene symbols as the first column
cpm_norm_out <‐ cbind(Symbol = rownames(cpm_norm), cpm_norm)
# Save normalized expression matrix for TRIAGEgene
write.table(cpm_norm_out, "TRIAGEgene_input_CPM.txt", quote = FALSE,
sep = "\t", row.names = FALSE)

  The resulting file, TRIAGEgene_input_CPM.csv, contains CPM‐normalized expression values and can be used directly as input for TRIAGEgene.
8 (Optional) Perform batch correction if required.


R
# Install and load limma if batch correction is required
if (!requireNamespace("limma", quietly = TRUE)) {
BiocManager::install("limma")
}
library(limma)
# Generate log2 CPM values for batch correction
log_cpm <‐ cpm(dge, log = TRUE, prior.count = 1)
# Preserve the biological condition of interest during batch correction
design <‐ model.matrix(∼ condition, data = metadata)
# Remove batch effects
log_cpm_bc <‐ removeBatchEffect(log_cpm, batch=metadata$batch, design=design)
# Add gene symbols as the first column
log_cpm_bc_out <‐ cbind(Symbol = rownames(log_cpm_bc), log_cpm_bc)
# Save batch‐corrected logCPM matrix for TRIAGEgene
write.table(log_cpm_bc_out, "TRIAGEgene_input_logCPM.txt", quote = FALSE,
sep = "\t", row.names = FALSE)

Batch correction should only be performed when batch effects are present and are not completely confounded with the biological condition of interest. The metadata file should contain a column named “batch” that specifies the batch assignment for each sample. It is worth noting that batch correction is performed on logCPM values rather than CPM values, because log‐transformed expression values are more appropriate for linear modeling and reduce the influence of highly expressed genes. The resulting batch‐corrected logCPM matrix can be used directly as input for TRIAGEgene.

## COMMENTARY

### Background Information

TRIAGE has been broadly applied across diverse biological contexts including studies in neurodevelopment, cardiac research, livestock muscle biology, and single‐cell transcriptomics to uncover key regulatory genes, and its predictions have been consistently supported by orthogonal datasets and downstream validation analyses (Afonso et al., 2023; Friedman et al., 2024; Kojic et al., 2021; Plaisance et al., 2023; Shen, Werner, Chiu, et al., 2025; Shen, Werner, Lukowski, et al., 2025; Wehrens et al., 2022). For instance, TRIAGE has served as an independent method to validate findings in cardiomyocyte maturation (Friedman et al., 2024; Plaisance et al., 2023) and neurodevelopment (Kojic et al., 2021). More importantly, its utility as a discovery tool across different species has been demonstrated in multiple studies, as exemplified by the identification of previously unrecognized roles for SIX3 in human endoderm differentiation and RNF220 in cardiopharyngeal development in Ciona (Shim et al., 2020). Expanding on this, Afonso et al. (2023) applied TRIAGE in bovine muscle samples to identify candidate regulators of mineral composition, including GRIN1 and GRIN2C (calcium) and HPCAL4 (iron). A subsequent analysis revealed that genetic variants of TRIAGE‐identified bovine genes, e.g., PITX2 and BTNL9, were associated with phenotypes linked to beef quality traits (Afonso et al., 2025). In the context of single‐cell transcriptomics, Shen, Werner, Chiu, et al. (2025) applied TRIAGE tools to scRNA‐seq datasets of induced pluripotent stem cells (iPSCs) mesendoderm differentiation and identified key regulators defining discrete cell populations within a continuum of heterogeneous cell states. Additionally, Wehrens et al. (2022) applied TRIAGE to identify regulatory candidates to distinct cell clusters in a hypertrophic cardiomyopathy study, facilitating deeper insights into disease‐associated cell type‐specific gene regulation. Collectively, these studies demonstrate utility of TRIAGE not simply in reproducing known biology, but also in discovering previously unrecognized genetic regulators across different species.

Beyond published studies, the TRIAGE toolkit offers broad potential for integration into bioinformatics workflows involving gene sets or genomic loci. For instance, TRIAGE can be paired with transformer‐based models such as Geneformer (Theodoris et al., 2023), which output candidate gene sets from scRNA‐seq data. TRIAGE may then be applied post hoc to prioritize biologically meaningful regulatory genes from these predictions. TRIAGE can also be paired with genome‐wide association studies to prioritize SNPs and genomic loci with biological relevance and regulatory potential. Conversely, TRIAGE‐derived genome‐wide RTS and ranked gene sets or genomic loci can serve as input features for machine learning model training or interpretation, establishing a feedback loop between computational prediction and biological inference. This interoperability makes TRIAGE a versatile and biologically grounded complement to a wide range of bioinformatics workflows.

Benefiting from its modular architecture and annotation‐independent framework, the TRIAGE toolkit supports a broad spectrum of experimental designs and data modalities. Each module is designed to perform a specific analytical function and can be applied independently or in combination, depending on the research context. This flexibility enables TRIAGE to support diverse use cases, including:

Case‐control studies, where TRIAGEgene and TRIAGEparser can be applied to both differentially expressed gene sets and custom‐selected gene lists to identify key regulatory genes and pathways.
Single‐ or multi‐condition studies, where TRIAGEgene can be applied to rank genes by their intrinsic regulatory potential, followed by TRIAGEparser for downstream pathway analysis of selected gene sets.
Single‐cell transcriptomic studies, where TRIAGEcluster refines cell clustering, followed by TRIAGEgene and TRIAGEparser to identify cluster‐specific regulatory genes and pathways.
Functional genomics applications, where TRIAGEccs enables the identification of regulatory and functional elements across the genome at single‐base resolution.

Together, these modules make TRIAGE suitable for integration into both traditional and advanced bioinformatics analysis workflows.

### Critical Parameters

For the TRIAGEgene analysis, input gene expression values should be pre‐normalized (e.g., CPM, FPKM, or log‐normalized counts). While TRIAGEgene can automatically detect whether log transformation is required, users should ensure that raw count data are not used directly without appropriate normalization. In addition, TRIAGEgene requires gene symbols as row names in the input matrix. Incorrect or inconsistent gene identifiers (e.g., Ensembl IDs without conversion) may lead to reduced overlap with the RTS reference, resulting in fewer genes being evaluated. The “species” parameter must also be correctly specified, as gene symbols differ between species.

For TRIAGEcluster and byPeak analyses, the expression matrix and metadata file must contain matching cell identifiers. The first column of the expression matrix should correspond exactly to the cell identifier column specified in the metadata file. Inconsistent or mismatched identifiers will result in failed or incorrect grouping of cells.

When running TRIAGEccs, the genome build of the input BED file must match the RTS genome file (e.g., hg38 or hg19). Using mismatched genome builds will lead to incorrect CCS calculations, so coordinate consistency should be confirmed prior to analysis. TRIAGEccs requires BED format input with at least three columns (chromosome, start, end). For exon‐level analysis (exons_only = TRUE), additional BED12 fields (blockSizes and blockStarts) must be present. Improperly formatted BED files may lead to errors.

### Troubleshooting

A troubleshooting guide for using the TRIAGE toolkit is provided in Table 2.

**Table 2:** Troubleshooting Guide for Using the TRIAGE Toolkit

Problem	Possible reason	Solution
No such file or directory	The working directory is not correctly specified	Use “setwd()” to specify the correct working directory and ensure that the input files are in this directory
The number of genes found in the RTS table is low when running TRIAGEgene	The species was incorrectly specified, or the row names of the data frame are not gene symbols	Use the “species” parameter to specify the correct species and ensure that the row names of the input data frame are gene symbols
Failure to extract normalized gene expression data when using “GetAssayData()”	Incorrect argument specified in the “GetAssayData()” function, depending on the Seurat version	In Seurat v5, the “GetAssayData()” function requires the layer argument (e.g., layer = “data”), whereas in Seurat v4 and earlier versions, the argument is slot (e.g., slot = “data”); ensure that the correct argument is used according to the installed Seurat version
Could not find UMAP columns or cell identifier column when running TRIAGEcluster	Incorrect parameter settings for the cell identifier column and/or UMAP coordinate columns	Ensure that the metadata file contains a cell identifier column and two UMAP coordinate columns and specify the correct column name for the cell identifier and the correct column prefix for the UMAP coordinates
“TRIAGE_hg38.bedgraph” does not exist or is unreadable when using TRIAGEccs	The TRIAGE bedgraph file was not downloaded, or the file path was specified incorrectly	Download the correct TRIAGE bedgraph file and specify its path using the “rts_genome” parameter
Failure to run TRIAGEccs with the “exons_only = TRUE” option	The BED file lacks exon information (requires columns 11 and 12)	To calculate CCS for exon regions, ensure that the BED file is in standard BED format and includes the 11th (blockSizes) and 12th (blockStarts) columns

### Understanding Results

In Basic Protocol 1, the primary output from TRIAGEgene is a data matrix with gene symbols in the first column and TRIAGE‐weighted DS values for each sample in the subsequent columns (step 7). For datasets with multiple biological replicates, a Jaccard similarity heatmap can be generated to assess consistency across samples (Fig. 2; step 8). Selected differentially expressed genes, such as those expressed at higher levels in Sham P1 versus Sham P56, can then be extracted and ranked using DS values. The top‐ranked genes are saved in a tab‐delimited file (step 10). Table 1 shows the top ten TRIAGE‐ranked genes identified in step 10, including seven transcription factors related to embryonic development and tissue differentiation (Sox17, Wt1, Hoxd8, Ebf3, Foxc1, Ebf2, Nr2f2), one growth factor (Igf2), and two regulators of Wnt signaling (Sfrp1, Rspo1). Their prominence in the ranking reflects their strong regulatory potential. Although demonstrated here using a mouse dataset, TRIAGEgene is broadly applicable to normalized gene expression data from diverse biological contexts (Afonso et al., 2023; Friedman et al., 2023; Kojic et al., 2021; Plaisance et al., 2023; Wehrens et al., 2022), enabling regulatory gene prioritization without requiring prior annotations.

For scRNA‐seq data, as illustrated in Basic Protocol 2, a standard Seurat analysis pipeline generates a UMAP to visualize distinct cell clusters (Fig. 3A; step 5). Application of TRIAGEcluster identifies biologically coherent cell groups, or TRIAGE peaks (Fig. 3B; step 9). Toolkit‐integrated functions such as byPeak can then be used to compute average gene expression per peak or any user‐defined cell group. This enables seamless integration with TRIAGEgene, which can then prioritize regulators within each cell group. For example, TRIAGEgene identified top‐ranked regulatory genes in TRIAGE peak 8 (Fig. 3C; step 12), corresponding to dendritic cells. Notably, several top‐ranked genes, such as GATA3 (a transcription factor) and GNAS (a key signaling component), are well‐characterized regulators of cell function and development. To further explore gene function within each cell group, TRIAGEparser can be used to group genes into gene clusters. Each gene cluster can be characterized using STRING GO enrichment analysis, with enriched GO terms visualized in a heatmap format (Fig. 3D; step 13). This modular workflow enables both targeted and global insights into regulatory gene activity at the single‐cell level.

In Basic Protocol 3, CCS values are computed for 5188 lncRNAs on human chromosome 12 using data from the LNCipedia database (step 3). Inflection point analysis identifies 723 lncRNAs as significantly cell‐constrained, and these are prioritized as regulatory candidates (steps 5 to 6). Among these, well‐studied regulatory lncRNAs such as HOTAIR (Gupta et al., 2010) (CCS = 0.9999) and antisense transcripts from the HOXC cluster (Zhou et al., 2020) were successfully recovered, validating CCS as an effective metric for prioritizing lncRNAs by regulatory potential. The primary output of this protocol is a tab‐delimited file (e.g., “LNCipedia_chr12_CCS.txt”), which contains genome coordinates and associated annotations for each lncRNA, with CCS values provided in the final column. This file enables ranking and downstream selection of candidate regulatory lncRNAs.

In Basic Protocol 4, TRIAGEccs is applied to prioritize functional variants from GTEx cis‐eQTL data in the human heart left ventricle. After computing CCS values for all variants, an inflection point is used to distinguish high‐ and low‐CCS variants. Subsequent analysis reveals that high‐CCS variants are significantly enriched for stronger regulatory signals. Specifically, high‐CCS variants exhibited substantially lower nominal *p*‐values [mean –log₁₀(*p*‐value) = 14.39] than low‐CCS variants (mean = 10.45), with a Wilcoxon test *p*‐value <2 × 10^−16^ (step 9). Additionally, effect sizes were significantly greater for high‐CCS variants (mean = 0.4811) compared to low‐CCS variants (mean = 0.3776) (step 10, *p*‐value <2 × 10^−16^, Wilcoxon rank‐sum test). These results confirm the utility of CCS in prioritizing variants with stronger phenotypic impacts. The primary output of this protocol is a tab‐delimited file “Heart_Left_Ventricle_variants_CCS.v8.all.bed”, which contains genomic coordinates for each variant, with CCS values reported in the final column to indicate regulatory potential.

Importantly, DS values (in Basic Protocols 1 and 2) should be interpreted as relative prioritization scores rather than absolute measures of regulatory function. They are most useful for ranking genes within samples, conditions, or cell populations that have been processed together using the same preprocessing workflow. Direct comparison of DS values across independently processed datasets, species, or unrelated gene sets should generally be avoided, because differences in data processing, including normalization, gene filtering, and species‐specific RTS references, may influence the score distribution. CCS values (in Basic Protocols 3 and 4) should be interpreted as relative prioritization scores for genomic loci analyzed using the same genome build. Direct comparison of absolute CCS values across different species or genome builds is not recommended, because these analyses rely on species‐ and build‐specific genomic coordinates and RTS reference files.

### Time Considerations

The TRIAGE toolkit is designed to be computationally efficient. All four Basic Protocols and the Alternate Protocol can be completed within 30 min on a standard personal computer. The toolkit has low memory requirements, and most analyses can be readily performed on a personal laptop. For single‐cell RNA‐seq analyses involving large numbers of cells, the upstream Seurat workflow may take several hours. In such cases, both Seurat preprocessing and TRIAGEcluster analysis are recommended to be performed on a high‐performance computing server.

### Author Contributions

**Qiongyi Zhao**: Conceptualization; methodology; writing—original draft; writing—review and editing. **Sophie Shen**: Methodology; writing—review and editing. **Yuliangzi Sun**: Methodology; writing—review and editing. **Enakshi Sinniah**: Methodology; writing—review and editing. **Mikael Boden**: Supervision; writing—review and editing. **Nathan Palpant**: Conceptualization; supervision; writing—review and editing. **Woo Jun Shim**: Conceptualization; methodology; supervision; writing—review and editing.

### Conflict of Interest

The authors declare that they have no competing financial interests.

## Data Availability

All data used in this study are publicly available. To facilitate the testing of the TRIAGE toolkit, we also provide the test datasets on GitHub at: https://github.com/Qiongyi/TRIAGE_R_Package_v2/tree/main/protocol. The TRIAGE toolkit is available through the UniQuest Online Store (https://uniquest.store/product/triage2) under either an Academic Research & Teaching license, which is free for academic research and teaching, or a General Use license agreement for other purposes. Its documentation is provided at: https://tinyurl.com/triage2doc.
